# Distributed dynamic strain sensing of very long period and long period events on telecom fiber-optic cables at Vulcano, Italy

**DOI:** 10.1038/s41598-023-31779-2

**Published:** 2023-03-21

**Authors:** Gilda Currenti, Martina Allegra, Flavio Cannavò, Philippe Jousset, Michele Prestifilippo, Rosalba Napoli, Mariangela Sciotto, Giuseppe Di Grazia, Eugenio Privitera, Simone Palazzo, Charlotte Krawczyk

**Affiliations:** 1 Istituto Nazionale di Geofisica e Vulcanologia-Osservatorio Etneo, Piazza Roma 2, Catania, Italy; 2 Department of Electrical, Electronic and Computer Engineering, University of Catania, Viale Andrea Doria, 6, 95125 Catania, Italy; 3 GFZ German Research Centre for Geosciences, Einsteinstrasse 42-46, 14473 Potsdam, Germany

**Keywords:** Natural hazards, Solid Earth sciences, Mathematics and computing

## Abstract

Volcano-seismic signals can help for volcanic hazard estimation and eruption forecasting. However, the underlying mechanism for their low frequency components is still a matter of debate. Here, we show signatures of dynamic strain records from Distributed Acoustic Sensing in the low frequencies of volcanic signals at Vulcano Island, Italy. Signs of unrest have been observed since September 2021, with CO_2_ degassing and occurrence of long period and very long period events. We interrogated a fiber-optic telecommunication cable on-shore and off-shore linking Vulcano Island to Sicily. We explore various approaches to automatically detect seismo-volcanic events both adapting conventional algorithms and using machine learning techniques. During one month of acquisition, we found 1488 events with a great variety of waveforms composed of two main frequency bands (from 0.1 to 0.2 Hz and from 3 to 5 Hz) with various relative amplitudes. On the basis of spectral signature and family classification, we propose a model in which gas accumulates in the hydrothermal system and is released through a series of resonating fractures until the surface. Our findings demonstrate that fiber optic telecom cables in association with cutting-edge machine learning algorithms contribute to a better understanding and monitoring of volcanic hydrothermal systems.

## Introduction

Volcanic hazard assessment requires geophysical, geochemical and geological information, which are recorded by a number of instruments deployed on the flanks and the summit of volcanoes. In particular, the nature of very long period (VLP) and long period (LP) seismic signals at volcanoes has been since several decades a matter of debate^1^. LP events are generally thought to be associated with fracture resonance^2–4^ or transport of fluids within the conduit and its surrounding^5^, but other explanations have been also proposed^6–8^. Their typical frequency content ranges from ~ 0.2 to ~ 5 Hz. They often precede and accompany volcanic eruptions^9^. VLP events are recorded at volcanoes with different eruptive styles^10–12^ and are associated with inertial movement of heat, gas and magma, and interactions between magma and subterranean water within the volcanic plumbing system^13^. Their typical frequency content is below ~ 0.2 Hz. Understanding the source mechanisms of LP and VLP signals is, therefore, a key component in assessing volcano unrest and providing early warning for ensuing eruptions. Broadband seismometers have been the main instruments to estimate source processes of LP and VLP signals so far at volcanoes^14^. They are usually deployed in arrays of instruments covering the volcanic edifice^15^ to catch the events and estimate their source^16^. However, on small volcanic islands, the lack of coverage in the submarine environment hinders our understanding of the volcanic sources, preventing accurate hazard to be assessed, especially when a crisis starts (e.g., Tonga eruption^17^). Yet, submarine instrumentation (such as Ocean Bottom Seismometers) is costly and may be used for temporary experiments^18^. Using DAS technology, fiber optic cables have been recently shown to be able to sense seismicity in volcanic setting^19,20^, in glacier environment^21^, and in submarine deployment^22^. Owing to the distributed feature of DAS measurements over long distances (tens of km), fiber optic sensing produces a huge amount of data, demanding for automated processing workflows. Recently, the development of data-driven techniques has been rapidly progressing under the impulse of advances in artificial intelligence and machine learning (ML). Within the ML field, especially the deep learning (DL) algorithms^23^ are rapidly evolving for directly extracting high-level features from large datasets without the intervention of human experts^24,25^. A number of applications on volcano-seismology for signal identification and classification have already been published^26,27^ showing the good performance of these techniques. Titos et al.^28^ successfully used recurrent neural networks to detect and classify continuous sequences of volcano-seismic events. More recently, Lara et al.^29^ have presented a quasi-real-time automatic recognition (detection and classification) of micro-earthquakes at Cotopaxi volcano based on a deep learning approach.

**Figure 1:**
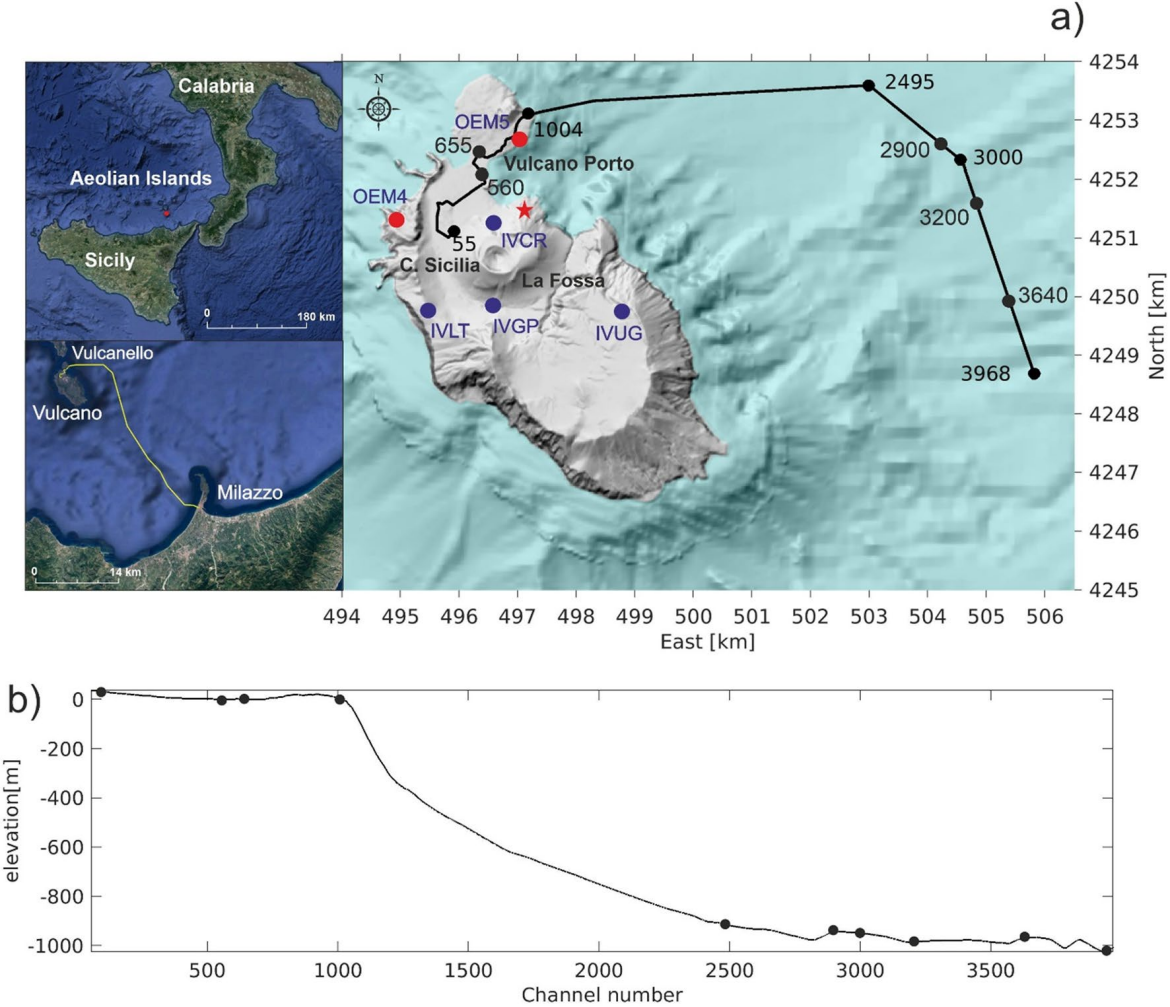
(**a**) Location map of TIM fiber optic cable connecting Vulcano to Milazzo interrogated by a DAS device hosted at the TIM power station of Vulcano. Permanent (IV*, blue dots) and mobile (OEM*, red dots) broadband seismic stations operating at Vulcano and used in the analysis are also reported. (**b**) Topographic and bathymetric profile along the fiber-optic cable route. Fiber channel numbers at specific locations are shown (black dots). The red star indicates the location of the source of the VLP events, obtained by a grid search method based on radial semblance function^30,51^.

Here, we characterize volcanic dynamic strain at very low frequencies (down to 0.1 Hz) with conventional algorithms and deep learning approaches, applied on DAS records from an existing telecom fiber optic cable linking Vulcano Island to Sicily (Italy). Vulcano is the southernmost of the Aeolian Islands, located at about 25 km from northern Sicily (Fig. 1^30^; Supplementary Information S1). In September 2021, outstanding changes in the rate and amplitudes of several geophysical and geochemical parameters, routinely monitored (temperatures, gas emissions, ground deformation, seismicity), marked a new unrest at Vulcano^31–33^. A substantial increase in temperature (up to around 380 °C at La Fossa crater) and flux of soil and fumarolic gas emissions were observed^32^. In particular, CO_2_ emission rate reached 34,000 gm^−2^ day^−1^ exceeding the background levels by several orders of magnitude in different areas of the island^31^. Also the SO_2_ flux in the plume emitted in the summit area exceeds the background values by one order of magnitude, reaching the maximum value of 240 t day^−1^^31^. In association with these geochemical anomalies, significant ground deformation was recorded from September to beginning of November 2021. A general areal inflation of the volcano edifice (about 22 ppm) was observed by GPS and DInSAR data^32^. The increase in gas emissions and ground deformation were accompanied by a significant seismicity, remarkable for the occurrence of amplitude transients with spectral peaks in the LP and VLP frequency bands.

Due to the seismic activity increase, the monitoring system managed by INGV on the island and especially on and around the active volcano has been expanded. In order to contribute to the increased monitoring effort associated with the crisis, we connected a DAS interrogator (iDAS® from Silixa) to an optical fiber cable, turning it into 3968 strain sensors with 4 m spacing (Fig. 1; “Methods” for details). From 13 January until 14 February 2022, we continuously recorded strain rate and we show that low frequency volcanic seismicity can be recorded all along the fiber.

Using conventional and deep learning-based approaches, we demonstrate that DAS can foster seismological observations to improve monitoring on volcano islands and to contribute for rapid response to volcanic unrest.

## Data inspection

**Figure 2:**
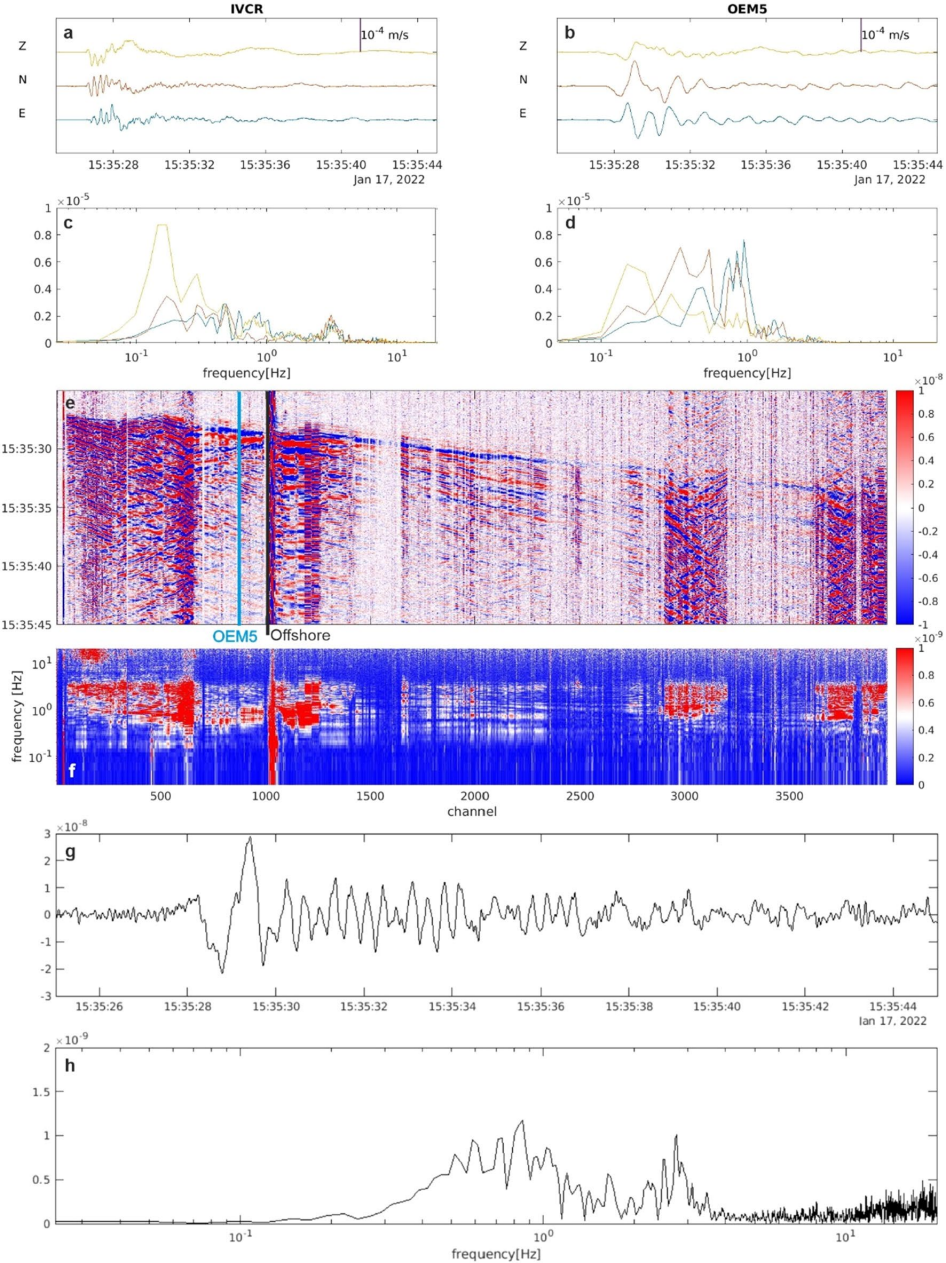
Representative example of a VLP signal recorded by the DAS and by the broadband seismic sensors at IVCR station, the closer to the crater area, and at the OEM5 station, the nearest to the fiber cable (see Fig. 1). (**a**,**b**) 3C seismic signals recorded at IVCR and OEM5 (near to the fiber channel 888; Fig. 1) and their spectra (**c**,**d**). (**e**) DAS strainrate signal filtered below 10 Hz. (**f**) Spectra of the DAS strainrate record. Traces of the DAS strainrate (**g**) and its spectrum (**h**) at the channel near to the OEM5 station.

**Figure 3:**
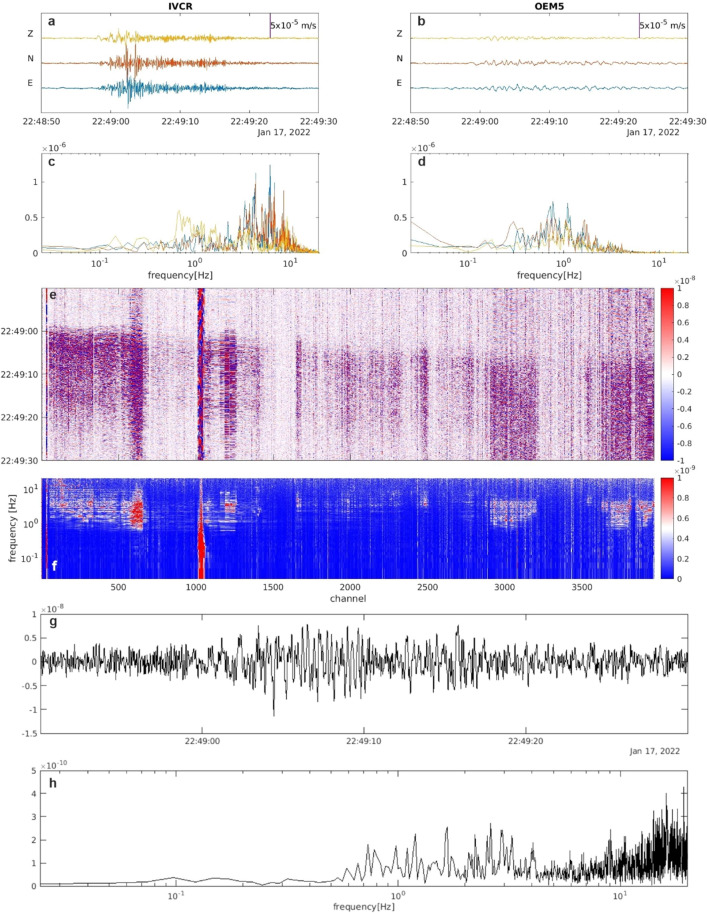
Example of LP event: (**a**,**b**) 3C seismic signals recorded at IVCR (in the summit area) and at OEM5 (nearest to the fiber) and their spectra (**c**,**d**). (**e**) DAS strainrate signal filtered below 10 Hz. (**f**) Full spectra of the DAS strainrate record. Traces of the DAS strainrate (**g**) and its spectrum (**h**) at the channel near to the OEM5 station.

**Figure 4:**
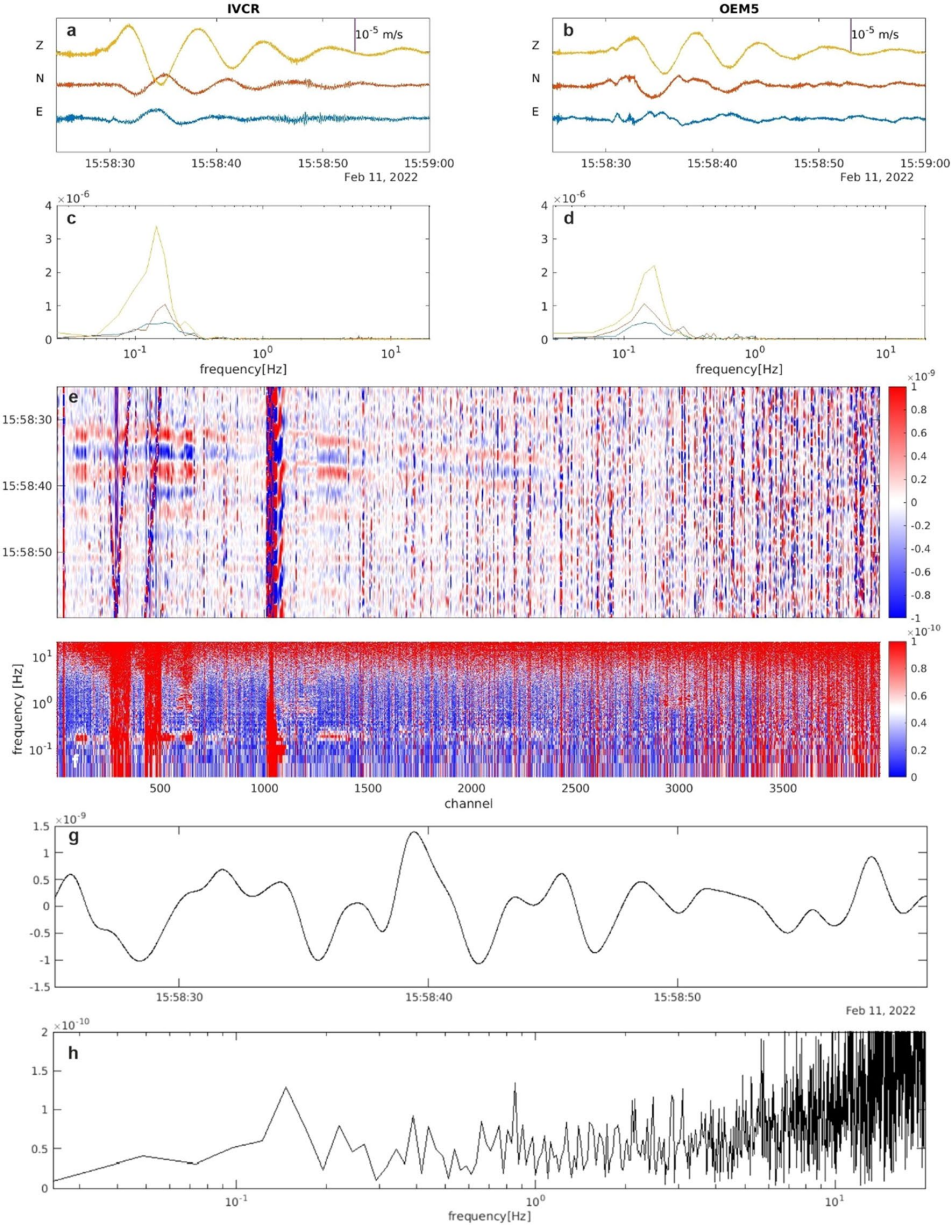
Example of a monochromatic VLP event: (**a**,**b**) 3C seismic signals recorded at IVCR (in the summit area) and at OEM5 (nearest to the fiber) and their spectra (**c**,**d**). (**e**) DAS strainrate signal filtered below 10 Hz. (**f**) Full spectra of the DAS strainrate record. Traces of the DAS strainrate (**g**) and its spectrum (**h**) at the channel near to the OEM5 station.

We observe varied dynamic strain responses along the fiber (Figs. 2, 3 and 4). The diverse signal-to-noise ratio (SNR) is most likely due to the highly variable mechanical coupling conditions between the cable and the ground both on land and off-shore. The cable runs through the urban area of Vulcano in pre-existing cased conduits (channels 50–1000), hindering a good coupling between the ground and the cable that instead could be easily achieved by direct-burial installation^5,34^. Off-shore (channels 1000–3968), the signals show peculiar localized features related to installation conditions. At shoreline (channels 1010–1055) the cable enters the sea inside a protective conduit and a persistent low frequency noise is observed, likely due to the impact of breaking waves approaching the shoreline. Two portions of the cables at channels 2900–3200 and 3640–3968 show a persistent oscillating signal with a peak frequency at about 1–2 Hz, similar to ringing noise observed in borehole installation^35^, which may be indicative of hanging of the cable in the water column between successive ridges of the bathymetry. A lack of signal is persistently experienced at some channels where the submarine fiber cable, just lying loose on the seafloor, may be decoupled from the seafloor^36–39^. Despite those loose signal portions, thanks to the highly dense spatial sampling of the DAS, we clearly identify low frequencies of the volcano seismic events down to 8–10 s on large sections of the submarine cable (Fig. 4).

DAS records allow us to follow the change in frequency and amplitude over distance, suggesting a potential use of DAS for studying site effect at Vulcano, that would be otherwise challenging using sparse seismic stations. An evident amplification of the signal is observed in correspondence to La Fossa-Vulcanello isthmus (channels 560–655), where the Vulcanello lava platform is joined to the main island of Vulcano through the progressive accumulation of pyroclastic products^40,41^. This is one of the main exhalative areas of Vulcano, characterized by the presence of a mud pool and hot water sources, where several hot wells have testified the existence of a vast thermal aquifer^42^. Lateral heterogeneities in local surface geology, as well as the presence of an aquifer, can be responsible for a site effect amplifying the strain response significantly.

**Figure 5:**
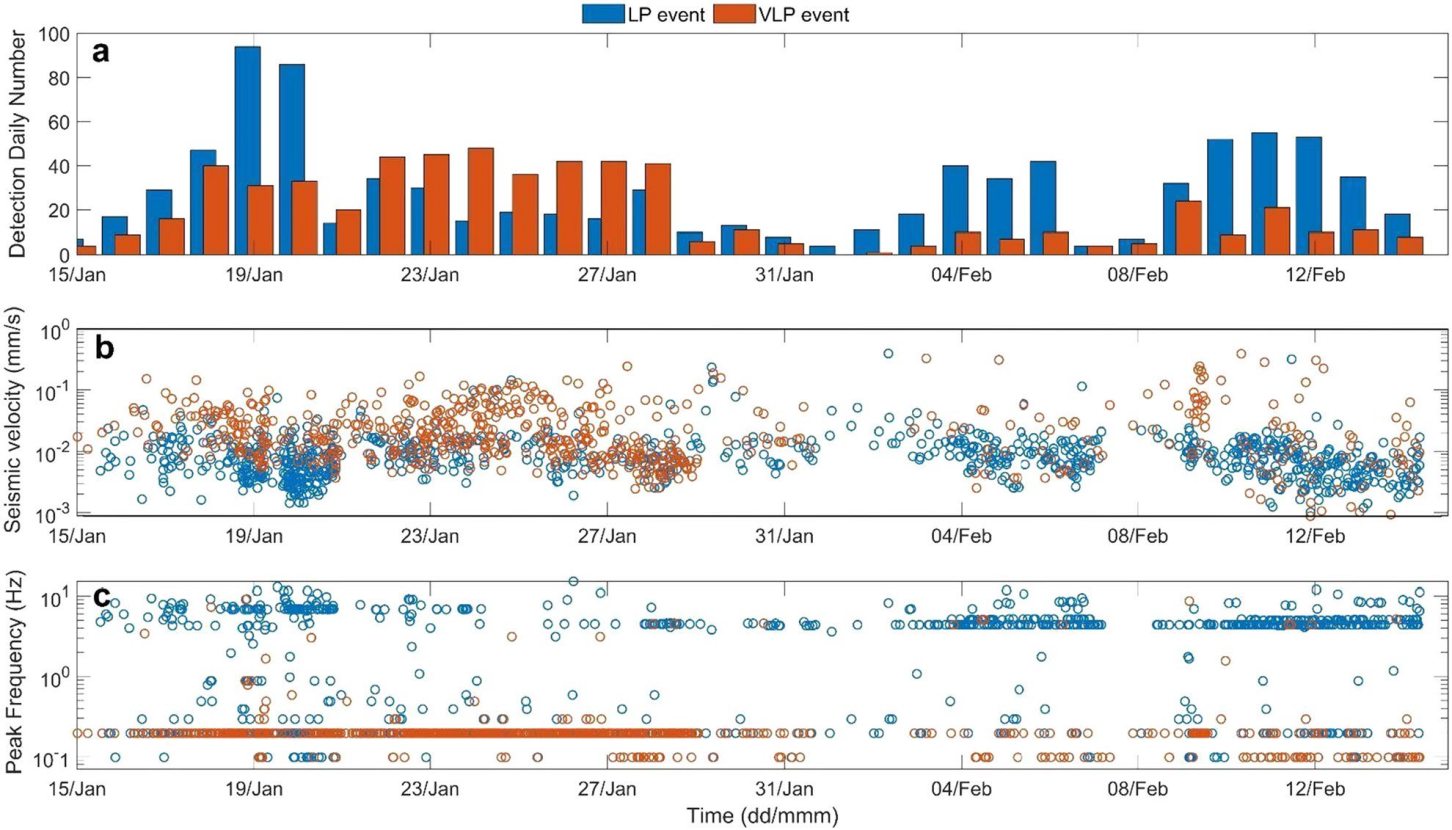
Daily number (**a**), peak-to-peak amplitude (**b**) and frequency peak (**c**) of LP (light blue) and VLP (orange) detected events of the INGV catalog in the time interval 15 January- 14 February 2022.

Figures 2, 3 and 4 show a variety of waveforms where multiple predominant frequency components are sometimes contemporaneously present. A preliminary data exploration allows us to distinguish three main classes of events. The first one is composed of LP events mostly in the frequency range 3–5 Hz and extremely up to about 8 Hz (Figs. 3 and 5). The other two classes show amplitude transients in the VLP frequency band and consist of a scarcely populated class of events with a monochromatic character (frequency peak centered at about 0.18 Hz, Fig. 4) and a class of VLP events with a broader frequency content spanning mainly from 0.1 to 6 Hz, thus also including an LP component (Fig. 2). The lowest frequency peak is centered at 0.18 Hz, as the monochromatic VLP events. Both types of VLP events are composed of decaying sinusoids lasting about 25 s.

The frequency content of both fiber optic data and broadband signal at OEM5 station (chosen for the comparison due to its vicinity to the fiber cable; Fig. 1a) are very similar (Fig. 2). The frequency content of the horizontal components at the OEM5 station differs significantly with respect to those recorded at the IVCR station, located closer to the summit crater area. At OEM5 station, the frequency peak at about 0.2 Hz, observed at IVCR, is attenuated and a peak around 1 Hz emerges as observed in the nearby DAS channel. On the other hand, the monochromatic VLP events preserve their waveforms at long distance (Fig. 4).

The higher frequency waveforms (Fig. 3) are greatly impacted by path effects, including scattering of waves from stratigraphic layering^43,44^, extensive fracturing^45^, topography^46^, and attenuation. In similar hydrothermal areas, Clark et al.^47^ have demonstrated that for distances farther than 1 km seismic waveforms and their frequency spectra may significantly be distorted by the presence of gas-saturated medium.

A simple comparison between seismic velocity at the broadband OEM5 station and DAS strain at the closest channel (Fig. S1) reveals a strong attenuation of the strain signal, which is even more weakened for the VLP events (Fig. 4). Not only coupling conditions, but also directional sensitivity of DAS could be responsible for this attenuation. Indeed, DAS measures the axial strain rate along the fiber and the result of the tensorial projection of the strain into the direction of the fiber cable implies that the amplitude of the DAS signal at each channel is controlled by the relative angle between the local cable direction and the passing seismic wavefront. Particle velocity and strain vary with the type of wave^48^. The VLP waveforms are similar on the three components of the seismometer records (Fig. 4) and, hence, the polarization is almost linear, along the propagation direction and points to the source direction like P-waves, as already observed for many VLP signals in volcanic areas^16,49,50^. The vast majority of the hypocenters of the VLP and the monochromatic VLP events was located below North of La Fossa Cone^33^, in the Forgia Vecchia area at a depth between 500 and 1500 m bsl^32^ (Fig. 1) and did not undergo variation in time. A consequence of the directional sensitivity of DAS is that the VLP waves, being at near normal incidence angle with respect to the cable, induce strainrate changes of a few nanostrain/s amplitudes in the DAS signals reaching the limit of the instrument resolution (1.4 nanostrain/s). However, the DAS noise level is low enough to distinguish the VLP event (Fig. S2).

## Events detection

We show that DAS can be used efficiently to detect low-frequency seismic events by adapting conventional seismic approaches and applying machine learning techniques. We compare the results with detections performed on conventional seismic signals. The analysis was performed from 15 January to 14 February 2022, when the DAS acquisition was continuous.

### Broadband seismic event detection

Due to the Vulcano crisis, an automatic system for event detection, characterization and source location on the broadband seismic signals has been implemented in the permanent monitoring broadband seismic system. In particular, we adapted the one developed on Etna for low frequency seismicity^51^. The elaboration system automatically runs on data in near real-time and implements an energy trigger algorithm (STA-LTA) on signals recorded at IVCR or OEM4 stations (Fig. 1). The system computes two STA-LTA values on the signal filtered in two frequency bands: LP (0.5–5.5 Hz) and VLP (0.05–0.5 Hz). In order to identify and trigger events in the LP frequency band, window-lengths for the STA (short time average) and the LTA (long-term-average) were set equal to 0.7 and 7 s, and a trigger threshold of 4 was chosen. As for VLP event detection, window-lengths are equal to 2 and 20 s and the trigger threshold equal to 4. Once detected, frequency peaks and peak-to-peak amplitudes are computed for all the events (Fig. 5).

During the investigated period, 1488 events were detected, 891 in the LP frequency band and 597 in the VLP one. The daily occurrence rate, the amplitudes and the frequency of the seismic events vary within the month of activity. In the time interval when the DAS experiment was carried out, the occurrence rate underwent an increase on 16 January 2022, reaching a medium level with respect to the September–October period^32^. This increase lasted for about two weeks, and in February seismo-volcanic event number decreased persisting on a low level^32,33^.

### Conventional detection algorithm on DAS

We applied a recursive STA-LTA trigger algorithm^5^, adapted to the DAS records by stacking the STA-LTA functions computed for each channel. As for the seismic data, the DAS data were filtered in the two frequency ranges of LP and VLP bands. After exploring diverse configurations, we attain the optimal lengths of the short- and long-term windows as those used in the analysis of the seismic broadband signals. We selected the channels with the highest SNR, which are those from channel 50 to channel 600, in the urban area. Thanks to the dense spatial acquisition and the stacking procedure on the STA-LTA functions over the channels, a robust detection is achieved. Although the DAS signal is on average weaker and noisier than the signal acquired at the IVCR station, positioned closer to the crater area, the channel stacking enhances the detectability and lowers the noise. This is also effective in avoiding false positives due to anthropogenic noise (e.g.: cars, pedestrians, engines), which is rich in this urbanized area.

To evaluate the performance of the detection, we compared the DAS detected events with the 1488 events detected on the seismic signal with respect to whom we computed the numbers of the true positive (TP), the false positive (FP) and the false negative (FN) and the associated classification metrics (see definition in Table 1)^52,53^. As for the conventional STA-LTA algorithm, an event is declared when the DAS stacked STA-LTA exceeds a fixed threshold. By means of the classification metrics, we measured the performance at several threshold values from 1.4 to 6 (Fig. S3). Threshold values of 2.8 and 2.2 maximize the F1-score (Table 1) in the LP and VLP band, respectively, and warrant a good compromise between the numbers of TPs and FNs.

**Table 1:** Performance of the detection algorithms estimated using the following metrics: true positive rate (TPR = TP/(TP + FN)) also known as recall; false discovery rate (FDR = FP/(TP + FP)); precision (Prec = TP/(FP + TP)); F1-Score (2*Prec*TPR/(Prec + TPR)).

Metrics	STA-LTA		InceptionV3		Optimized CNN		
	LP	VLP	Test set	Continuous data	Test set	Continuous data	Unseen continuous data
TPR	0.75	0.50	0.89	0.97	0.84	0.94	0.75
FDR	0.24	0.20	0.07	0.17	0.06	0.13	0.17
Precision	0.75	0.81	0.93	0.83	0.94	0.87	0.83
F1-Score	0.75	0.62	0.91	0.89	0.89	0.90	0.79

We investigate the characteristics of the FP and the FN events. We observe that most of the FP events fall in the LP band and belong to small events that are difficult to be detected by a single measurement. On the other hand, the FNs correspond to small amplitude VLP events for which conventional power detectors, such as the STA-LTA algorithm, are not performing well. About 93% of the seismic events have an amplitude in the vertical component at IVCR station below 0.05 mm/s. The distribution of TP and FN shows that the FNs correspond to those events with amplitude lower than 0.01 mm/s (Fig. S4). Most of the FNs belong to the small amplitude monochromatic VLP events.

In order to enhance the signal detectability of these lower amplitude events, we run a 2D template matching on the DAS data. Signals filtered below 0.3 Hz are cross-correlated with a 2D template extracted among the largest monochromatic events and recorded on 4 February 2022 at 01:23 UTC. A 2D correlation coefficient is computed between the 20 s long template and the full dataset. Since the template is short and filtered in a narrow frequency band, it ensures a large correlation coefficient. An event is declared when the 2D correlation coefficient exceeds a threshold. As done for the STA-LTA detection, we optimized the threshold value by maximizing the performance measured by the F1-score. The threshold was varied in the range 0.4–0.95. The F1-score curve shows a better performance for a threshold of 0.7 (Fig. S3). Under this condition, we compared the 413 DAS detections with the 597 VLP events detected using the seismic network (Fig. S5). Exploring the characteristics of the false events, we observed that the FP events are indeed low-frequency seismic events, not detected by the automatic detection system run by INGV-OE. Therefore, the VLP events are better detected in the DAS signal using a 2D template matching, whereas they are too small in amplitude to be detected by the STA-LTA.

### Machine learning based event detection on DAS

We investigate whether it is possible to obtain a better detection accuracy with ML algorithms by comparing the results with the ones obtained from the conventional signal processing methods. Beside common ML approaches already proposed in some applications^54,55^, DL approaches are increasingly used for event detection using fiber sensing, as described in^56–58^. A major advantage of deep neural networks lies in their ability to extract relevant features from raw data in a hierarchical manner without requiring domain knowledge from the experts. In addition, deep neural networks provide a unified approach for selecting features and classifying them, removing the need to search for optimal classifiers.

Although there are already applications of ML and DL approaches for event detection in DAS data in industry and structural health monitoring^59^, to our knowledge, there are not so many attempts in their use for volcano monitoring. Just recently^29,60^, DL are becoming common tools for investigating and monitoring seismic and volcanic areas exploiting the spatially and temporally large DAS data, that may contain signals from natural and anthropogenic sources.

The deep learning approach is based on the idea of converting DAS signals into a sequence of images on which deep neural networks are applied to detect seismo-volcanic events. For this purpose, we trained two Convolutional Neural Network (CNN) architectures: Inception-v3 and an optimized CNN (see “Methods”).

We used supervised machine learning, which provides a label for each training instance. The raw DAS signals are firstly filtered below 5.5 Hz and down-sampled to 20 Hz to remove unwanted noise and decrease the dimensionality of the input data. Taking inspiration from computer vision, the large DAS data is divided into smaller matrices, miming a sequence of images, each representing a collection of signals recorded along the fiber at a certain time interval. In particular, we extract two-dimensional time windows (channel x time) from the filtered data and we label the extracted window either as (1) if they contain a target event, or as (0) otherwise as random noise. The considered target events consist of the seismic events detected on the seismic broad-band signal. After computing the typical day (i.e. average day calculated stacking all the days over the entire dataset), random noise windows were randomly extracted from time slots with the highest average noise, keeping them at least one minute away from the closest seismic event. The data windows of both classes have a size of 551 × 160, as they contain the recordings of the 551 channels with the highest SNR for 8 s. Each window was converted to a grayscale image (i.e., image with 1 color channel) and normalized in the range [− 1, 1].

Data pre-processing from 15 January to 14 February produced a dataset of 148,800 images of which 1% including volcanic events (1488) and 99% background noise (147,312). To train and validate the neural networks, the dataset was divided into training set (70%), validation set (20%) and test set (10%) maintaining the same imbalance between classes in the initial dataset.

After training, the two architectures achieved good and similar results. In terms of F1-Score, both of them reach 0.999 on the training set and 0.998 on the validation set.

Following the approach used in Huot et al.^60^, we then evaluate the trained networks both on the test set and on the continuous data. In the latter case, the neural networks are tested on the continuous data by extracting consecutive windows of size 551 × 160 from 15 January to 14 February. Then, the 345,101 windows were transformed into grayscale images. Table 1 reports results from both the test set and the continuous data in terms of classification metrics.

It should be noted that we did not remove the samples containing the events used in the training, because the continuous sampling procedure does not produce event-synchronized samples like those used in the training phase. Thus the continuous dataset does not contain any identical samples used in the training. In the continuous dataset, the duration of the events to be detected is variable and a single event may fall on multiple images that the neural network can all classify with label 1. Thus, in performance evaluation on continuous data, an event is considered correctly classified if the neural network predicts label 1 to at least one image in the time range of ± 10 s from the picking time of the declared seismic event. Results on the continuous dataset are reported in Table 1, for both the networks.

As a further check to assess the quality of the DL model, we temporally split the whole data into two subsets. The data until 10 February were used to train and validate a network with the same architecture as Optimized CNN, which was then tested on consecutive windows extracted from 11 February onwards by applying the method described above for continuous data. The temporal subdivision ensures that no information provided during training influences the test performance. Metrics on these unseen continuous data (Table 1) confirm the good results.

**Figure 6:**
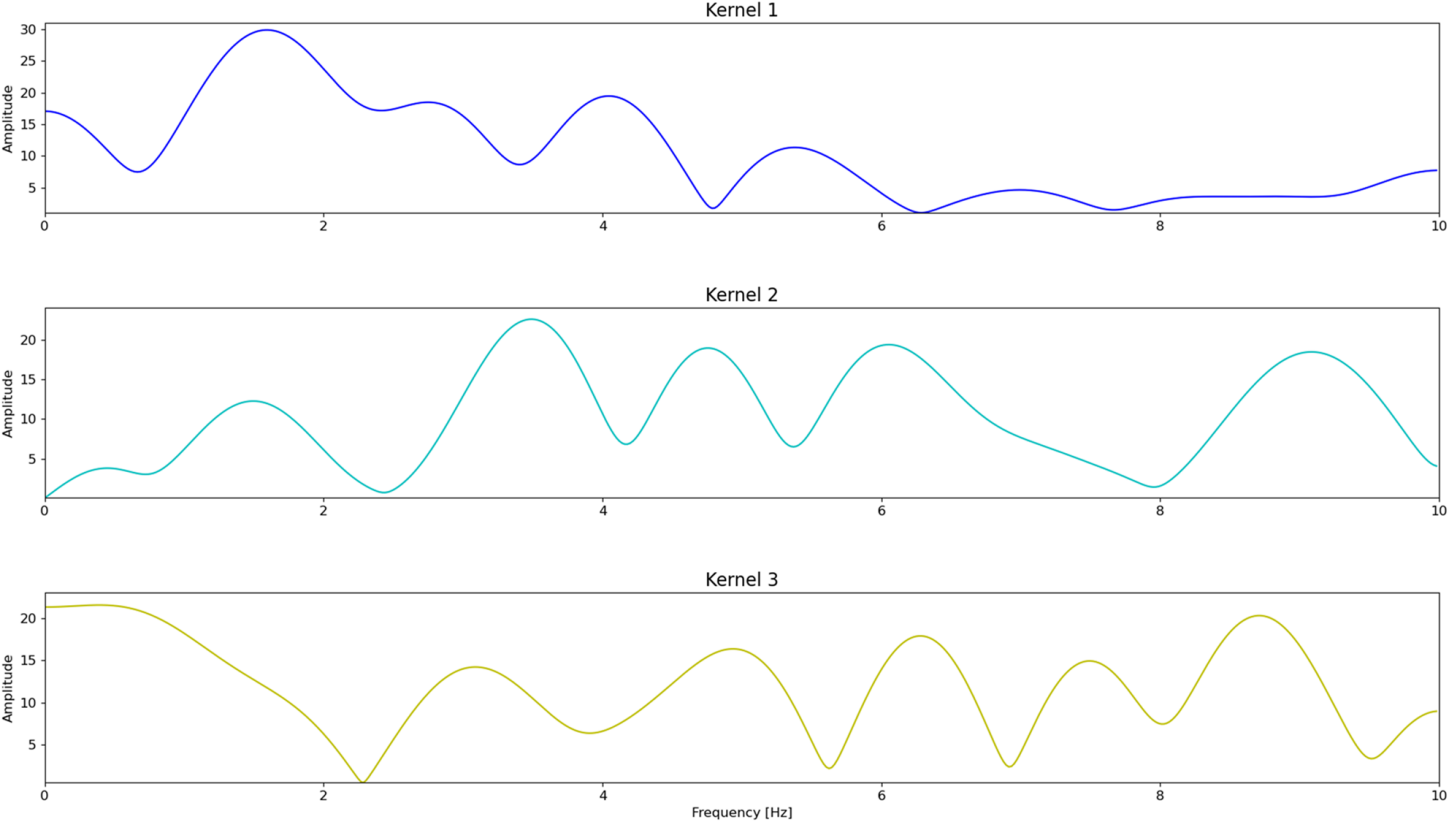
Frequency responses of the 3 kernels in the first convolutional layer of Optimized CNN network. Frequencies above 5 Hz are not significant because the signals were previously filtered below 5.5 Hz.

**Figure 7:**
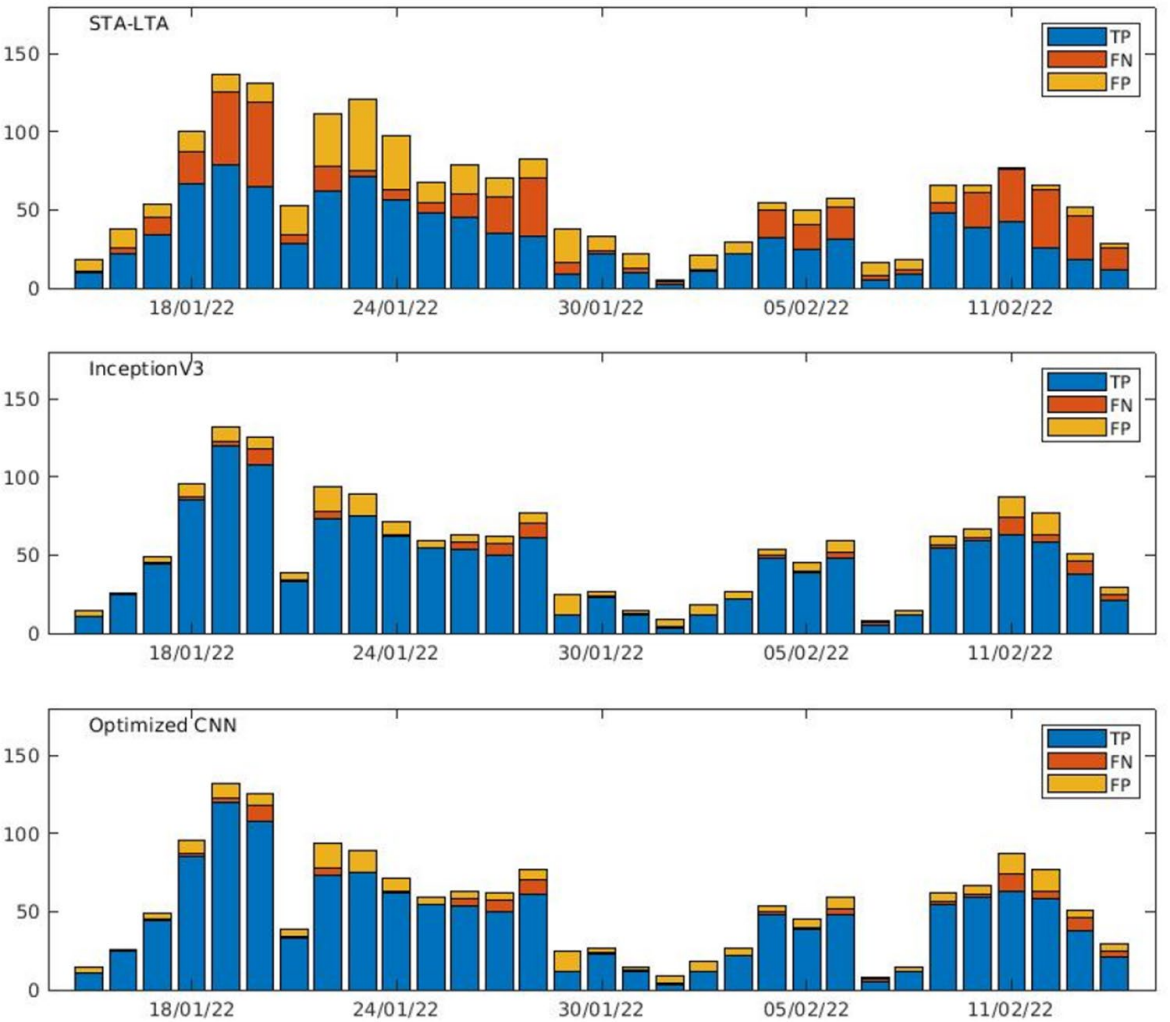
Distribution of daily numbers of DAS detected events using STA-LTA algorithm, the InceptionV3 and the optimized CNN.

The comparison of the metrics shows that ML techniques for the two architectures significantly outperform the conventional STA-LTA algorithm on the DAS dataset. As DAS images are fed into the deep learning architecture, the network is able to learn the representation useful for the detection. Although Optimized CNN is much shallower than Inception V3 (1,158,852 parameters the former and 24,348,324 the latter), it comes up with similar performance by leveraging the first kernels to emphasize low, mid and high frequencies (Fig. 6). In evaluating the results, it should be stressed the substantial difference between the adopted approaches. The performance of the STA-LTA algorithm is largely affected by the frequency band of the filter applied on the signal as well as by the optimal tuning of the window-lengths and the threshold. On the other hand, thanks to the ability in automatically extracting features from the training samples and generalizing from them, the DL enables to predict the pattern of unseen data facilitating the pre-processing steps. Figure 7 allows us to compare the daily distributions of the TP, FP, FN events obtained by the STA-LTA and the CNNs over the continuous dataset. The number of FNs in both CNNs is significantly reduced, denoting a better ability in the detection of the events. Notwithstanding the good performance of the networks, we observe a significant number of FPs, which correspond to subtle events not detected by conventional methods applied on a single seismic signal. By detecting more small events, ML approaches perform better than traditional approaches in conditions with lower SNR. On average, the detection performance of the algorithms over time is stable. The daily number of detected events drastically drops on 1–2 and 7–8 February. The detectability in both DAS and seismic signals is hampered by the high background noise, which is highly variable and sensitive to the meteorological conditions. In particular, an enhancement of the microseism was observed in those days (Fig. S6). Since the microseism spectral content (0.05–0.5 Hz) overlaps partly with the frequency band of the VLP events, the seismic signal is masked and difficult to detect.

## Events classification

**Figure 8:**
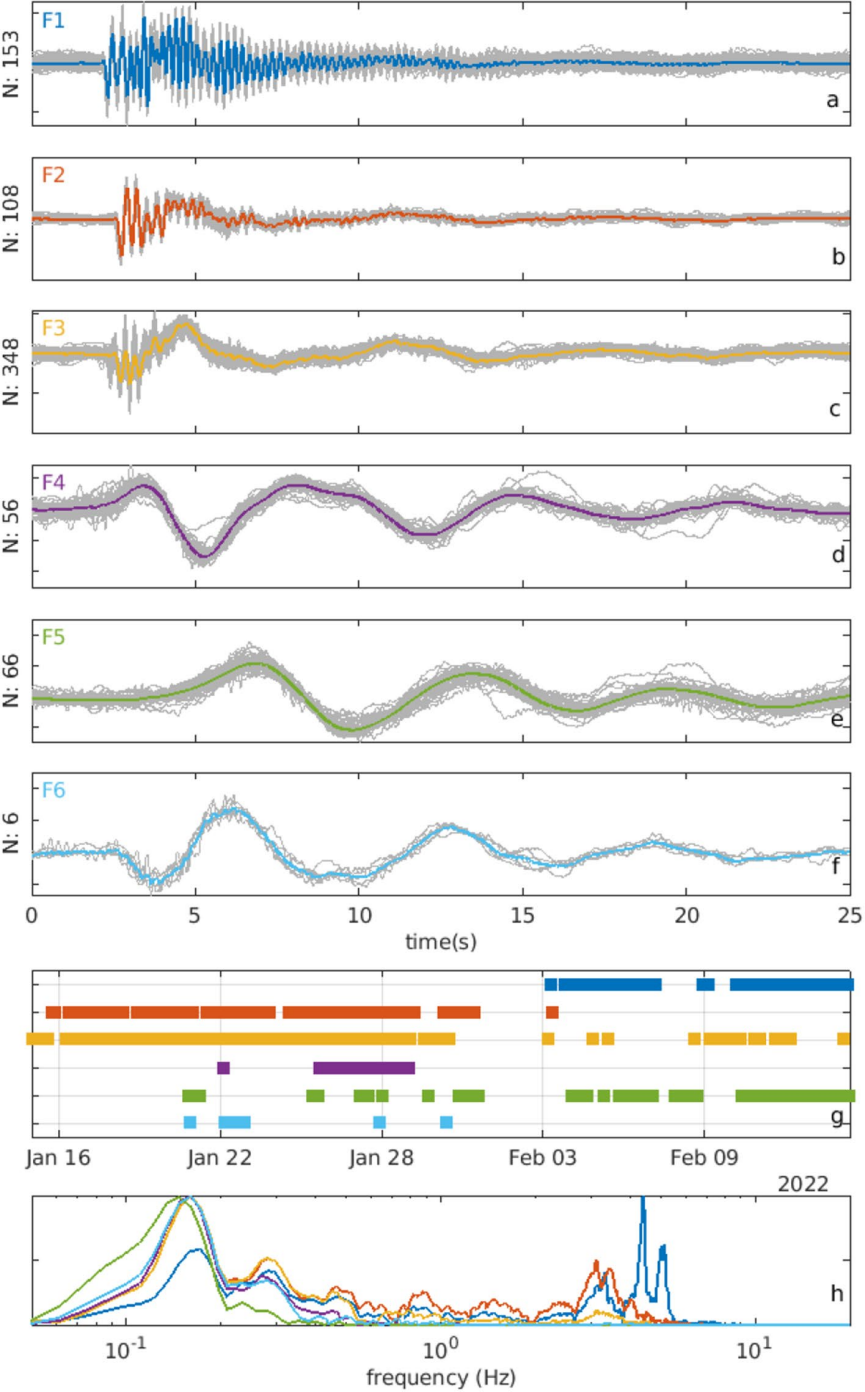
Stacked waveforms (thick coloured lines) of individual normalized events (thin gray lines) for the six identified families recorded at IVCR station (**a**–**f**). Each signal is normalized by centering the data to zero mean and scaling them to unity standard deviation. Temporal distribution of the occurrence of the IVCR classified events (**g**). Normalized spectra of the stacked waveforms (**h**).

**Figure 9:**
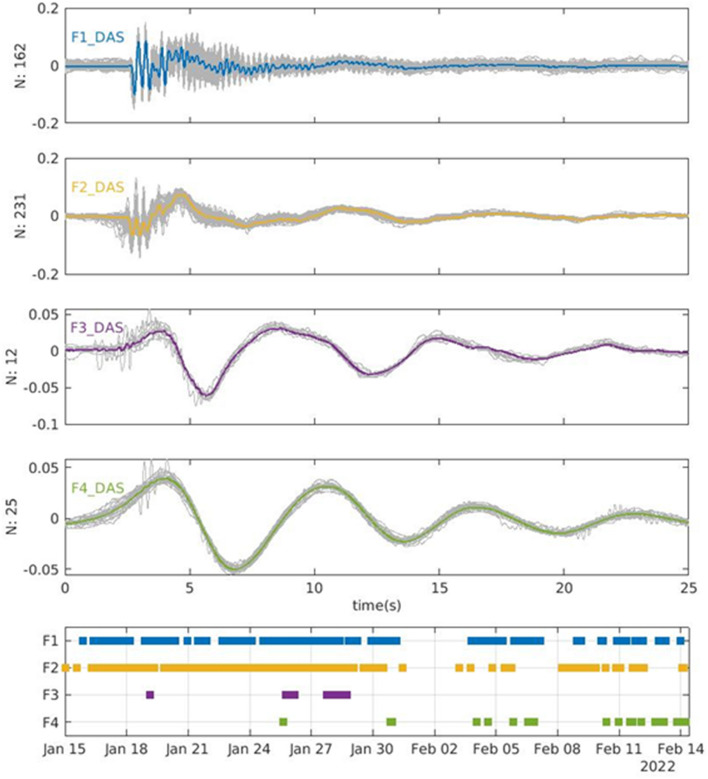
Stacked waveforms (thick colored lines) of individual normalized events (thin gray lines) for the identified families on the DAS data. The waveforms are those recorded at IVCR station to allow a comparison with the families identified using the seismic signal at IVCR (Fig. 6). Temporal distribution of the occurrence of the DAS classified events.

In the investigated period we observe a persistence of LP and VLP events which exhibit a strong waveform similarity and a mixed frequency content. To study their waveforms and occurrence, we perform a seismic event classification by computing the maximum cross-correlation between the pairs of the detected events, on both IVCR seismic data and DAS recordings. For the seismic IVCR data a time cross-correlation on a 11 s length window encompassing the event is used. For the DAS data a 2D cross-correlation on a similar window length of 11 s is used on the channels with the highest SNR on land. We then carry out a hierarchical clustering analysis applied successfully in volcanology^16^, using the Eardley algorithm^61^. We define here a family when a cluster contains more than 4 events. The build-up of the families depends on the selected cross-correlation threshold. We perform several tests by varying the threshold value and computing the number of distinctive families. Under a threshold of 0.9, the analysis on the IVCR cross-correlation matrix reveals six families (Fig. 8). The cluster analysis on the DAS cross-correlation matrix discloses similar waveforms, even if the number of families and associated events are lower (Fig. 9). Therefore, even if the waveform is distorted going from the summit area to the flank of the volcano (Figs. 2, 3, 4), the distinctive characters of the events are preserved and distinguishable. However, the families F1 and F2 recognized separately in the IVCR station are not discernable in the DAS data and are merged into only one family F1_DAS_. Similarly, the IVCR family F6 is not identified among the events on the DAS data. Indeed, the stacked waveform of the F6 family is very similar to the long wavelength component of the F3 family, showing a down-going onset followed by a sinusoidal oscillation. This evidence is confirmed by the high cross-correlation of 0.93 found between the F6 stacked waveform and the low-pass filtered signal (below 0.5 Hz) of the F3 stacked waveform. Indeed, excluding the family F5 and F1, high absolute correlation values (above 0.93) are found among the low-pass filtered waveforms of the families. Also the spectra confirm the strong similarity of the family F2-F4 and F6 in the low-frequency component. The waveform and the spectra of the stacked signal allows us to better distinguish and identify the three main types of events already noted from the preliminary data inspection. The classification clearly displays: (i) two higher frequency families (F1 and F2); (ii) a family with mixed frequency content (F3); (iii) three lower frequency families (F4, F5, F6). The family F1 shows two predominant frequency peaks at around 4 and 5 Hz, whereas in the family F2 the two peaks are shifted across a lower frequency of 3 Hz. Family F3 has two peaks at 3 Hz and at 0.18 Hz. The amplitudes of the higher and lower components are variable over time and give rise to a set of diverse waveforms. The monochromatic VLP events have distinctive characteristics with initial positive and negative polarities. F4 and F5 differ mainly at the onset of the event, when the F4 exhibits a faster evolution. Moreover, an intriguing strong negative correlation of − 0.97 is observed between the stacked waveforms of the events belonging to F4 and F6, which indicates a strong reversed polarity between the two families.

Temporal evolutions in the family distributions are observed. Particularly, at the beginning of February we observe a change in terms of occurrence and/or frequency of events in the families. This change occurs soon after a heavy rainfall (Fig. S6) that might have affected the seismicity by perturbing the pore pressure in fluid-filled fractures embedded in the hydrothermal system^62^. Owing to the short period of observations (one month) with respect to the longer duration of the crisis, inferences on the source mechanism could be not exhaustive. However, the distinctive features revealed by the classification analysis are indicative of their mechanisms. On the basis of our findings and similar evidences in other volcanoes^63–65^, we suggest a process of “breathing” of the hydrothermal system, in which the VLP component, mainly in families F6 and F3 and to a lesser extent in F2, could be related to its deflation by gas release and the LP component in families F1, F2 and F3 could result from gas escaping toward the surface through a series of resonating fractures. Only a few monochromatic VLP events report about the inflation of the hydrothermal system. The continuous gas emission since the beginning of September 2021 and the absence of any sign of deflation after the fast inflation phase in September–November 2021^32^ support the presence of a pressurized hydrothermal system that releases fluids through fractures toward the surface.

## Concluding remarks

Our results demonstrate that the existing telecommunication cable at Vulcano Island can be exploited to complement the seismic monitoring by using DAS technology. Owing to the Vulcano crisis we had the extraordinary opportunity to test the fiber cable, assess its seismic response and experiment traditional and innovative ML algorithms on the DAS dataset. Although the geometry of the telecommunication fiber cable and its coupling with the ground are not optimal to sense low frequency volcano seismicity, signatures of the seismic events are very well recognized in the DAS signals, also in the submarine section of the cable. We observe very low amplitude signals at the resolution limit of the instrument. However, the weakness of the signal is counteracted by the higher spatial density of the observations that allows for robust detection.

We exploit the distributed sensing feature of DAS measurements by adapting conventional algorithms and by re-designing ML approaches for image processing. The detection performance of the CNN networks on the continuous data suggest that, after initial training, the machine learning approach could be efficiently used for near real-time monitoring. While conventional algorithms required a fine tuning of parameters and filtering in appropriate bands in order to achieve a sufficient detectability, the deep learning-based event detection, without any a priori information on the frequency content of the events, performed better. In particular, the optimized CNN automatically adapts the frequency responses of the trained kernels for capturing and emphasizing the content of the signals at high, low and mixed frequencies. We find that the frequency content of most events comprises two distinct frequency bands. The classification analysis groups the events in families distinguished by the relative amplitude between the LP and the VLP components, suggesting they are generated by two distinct sources, the “breathing” of the hydrothermal system and a series of resonating fractures. Therefore, the fiber optic interrogation of telecom cable in association with dedicate processing algorithms can effectively contribute to the understanding of the low frequency seismic signals generated by the hydrothermal activity and, hence, may contribute to the rapid responses to volcanic crisis.

## Methods

### Distributed acoustic sensing experiment

From 13 January until 14 February 2022, we interrogated a 40 km-long submarine telecommunication fiber optic cable linking the TIM Vulcano power station to Milazzo (Fig. 1). The cable consists of 12 G652 fibers (single mode), of which 8 are used for telecommunication services. We connected an iDAS interrogator to one of the left 4 dark fibers and we applied a fixed gauge length of 10 m. We recorded strain rate data with a sampling frequency of 1 kHz and a spatial sampling of 4 m, turning the fiber into an ultra-dense array of 3968 sensors, also called “channels”. The cable passes through the urban area of Vulcano Island for about 3.9 km in pre-existing conduits (channels 50–1000) at a depth of about 0.8 m. Then it dives into the sea for about 37 km (channels 1000–3968) reaching a maximum sea depth of about 1000 m. At the shoreline (channels 1010–1055) the cable enters the sea inside a protective conduit for about 100 m and then is anchored to the volcanic rocks at distinct locations until it reaches the sandy seabed, where the cable is lying loose on the seafloor. Records exhibit a signal-to-noise ratio decaying over the distance, with a large noise beyond 20 km. Therefore, we acquired data until about 16 km distance up to channel 3968. A total dataset of 20 TB was collected.

After the installation, we performed hammer shots along the fiber path (provided by TIM) to geo-locate the DAS channels on land^19^ with a portable GPS device. For the submarine part, we interpolated the DAS channel positions from the cable locations, recorded at time of its installation, over a 20 m resolution bathymetry.

We compare the DAS data with seismic records from the land-based monitoring system managed by INGV-OE (Fig. 1). We use data from the four seismic stations belonging to the permanent network, equipped with broadband 3-component Trillium (Nanometrics) seismometers with cut-off period of 40 s, and two stations belonging to the stand-alone mobile network, installed in October 2021 to strengthen the seismic monitoring on the Island and equipped with 3-component Guralp 6 T broadband sensors with a cut-off period of 30 s. Stations of both networks acquire in real-time at a sampling rate of 100 Hz and data is transmitted to the operational room located in Catania.

From the monitoring seismic stations, most seismic signals are below 40 Hz. To fasten the data access and processing, the original DAS full dataset, after filtering below 50 Hz, was down sampled to 100 Hz and saved in HDF5 format.

### Deep neural model

**Figure 10:**
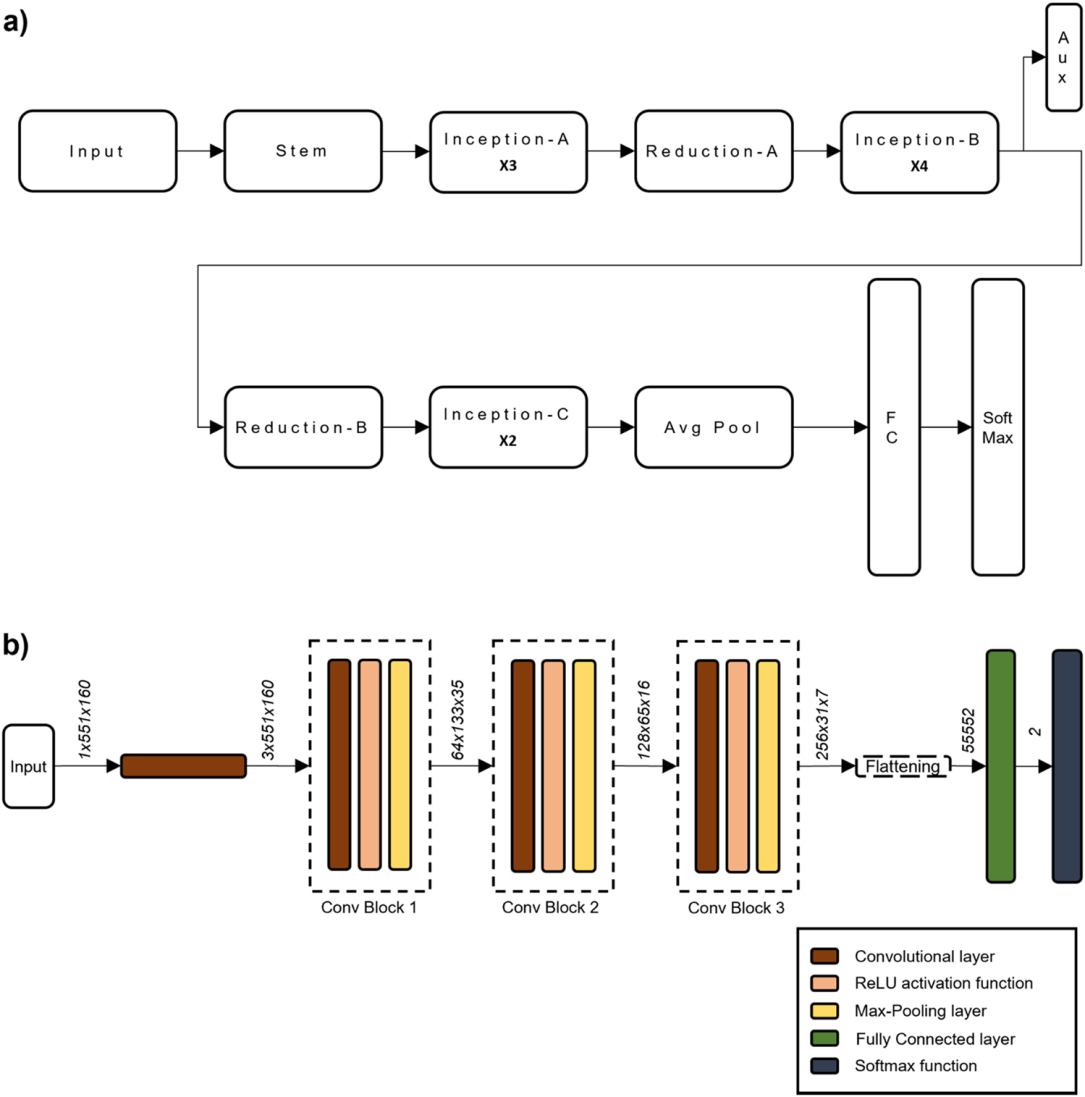
(**a**) Inception-v3 architecture. It includes three types of Inception modules, two of which perform the factorization of convolutions, both in smaller (Inception-A) and asymmetric convolutions (Inception-B), while the third type expands the filter bank outputs to promote high dimensional representations (Inception-C). This CNN exploits an auxiliary classifier (AUX) as a regularizing element and an efficient grid size reduction (Reduction-A and Reduction-B) by concatenating the feature maps obtained from convolutions with stride 2 and from max-pooling operation. Each convolutional layer is followed by batch normalization and Rectified Linear Unit (ReLU) activation function. (**b**) Optimized CNN architecture. The output tensor dimension of each layer (marked in Italics) is in the form number of channels, height, width.

We frame the detection as a binary classification between images containing or not seismo-volcanic events, and we tested two convolutional neural network (CNN) architectures, the Inception-v3 (Fig. 10a) and the optimized CNN (Fig. 10b).

#### Inception-v3

Inception networks^66–68^ mark a key milestone in image analysis. To overcome the extremely large variation in size of the salient parts in the image, an inception network uses filter banks with multiple dimensions operating on the same input level called inception modules. Specifically, Inception-v3 showed its quality for image classification in ILSVRC (ImageNet Large Scale Visual Recognition Competition)^69^.

For the event detection task, the Inception-v3 model available on Pytorch^70^ was instantiated. The architecture allows the network to process RGB images (i.e. images with 3 color channels) with height and width of at least 299 pixels and to predict one of 1000 classes. For our purpose, the model remained almost unchanged, except for the number of input channels of the first layer (from 3 to 1, for processing gray-scale images) and the output size of the two fully-connected layers (from 1000 to 2 classes). After resizing images to 551 × 299 (channel × time), data augmentation was performed on the dataset with random horizontal flipping and random vertical flipping. The network was trained on the training set by minimizing the Cross-Entropy loss function^71^ with mini-batch gradient descent using the Adam optimizer^72^—learning rate: 0.0001—and batch size 32, for a total of 50 epochs. The optimal network is the one that maximizes the F1-Score metric on the validation set.

#### Optimized CNN

The implemented convolutional network^73^ (Fig. 10b) consists of 3 convolutional blocks, each consisting of:

- A 2D convolutional layer, consisting of a set of filters (kernels), each of which is convolved with the image and creates an activation or feature map. The input (Inp) with dimension (*C*_*Inp*_*, H*_*Inp*_*, W*_*Inp*_) is transformed into output (Out) with dimension (*C*_*Out*_*, H*_*Out*_*, W*_*Out*_) by *K* kernels with *K* = *C*_*Out*_ with dimension (*C*_*Inp*_*,k,k*), where *C*_*Inp*_ is the number of channels and *k* is the dimension of kernel height *H* and width *W*.
- A rectified linear unit activation function, which receives as input the output of the convolutional layer. It maps negative values to zero while keeping positive values as they are, so as to produce *max(0,x)* as its output.
- A 2D max-pooling layer, reducing the height and width of the input while leaving the depth (i.e., the number of channels) unchanged. Each block applies independently *2* × *2* filters with stride of 2 (i.e. the step by which the filter is moved over the image) on every depth slice of the input, returning the maximum of each region.

The first block is preceded by a convolutional layer of 3 mono-dimensional kernels, each one with 21 parameters along the time axis (i.e., the width). The setting of the first layer allows the network to capture the signal dynamics at different frequency bands. Indeed, the frequency responses of the trained kernels (Fig. 6) show that they act as FIR filters by emphasizing the low (kernel 3), medium (kernel 1) and high frequencies (kernel 2).

Lastly, a fully connected layer and a SoftMax function^74^ classify the images into two classes: 0: 'no events', 1: 'event'.

#### Hyperparameter setting

We define the CNN network architecture by optimizing the combination of hyperparameters from a search space through the hyperparameter tuning process.

The optimization mainly regards the hyperparameters of the convolutional layer in the first block, the learning rate and the batch size. The search space is defined by values broadly used in literature^73^ as follows:

- Number of channels produced by the convolution in the first block *C*_*Out*_ ∈ {16,32,64}.
- Size of the convolving kernels in the first block *k* ∈ {3,5,7,9,11}.
- Stride by which the filter is moved by over the image *s* ∈ {1,2,3,4,5,6}.
- Zero-padding added to all sides of the convolutional layer input in the first block p ∈ {1,2,3,4,5,6}.
- Dilation between kernel elements of the convolutional layer in the first block *d* ∈ {1,2,3,4,5,6}.
- Learning rate *lr* ~ U(0.0001,0.1).
- Batch size *B* ∈ {32,128}.

Furthermore, convolutions of the second and third blocks double the number of channels of their own input; therefore, the depth of the output of each subsequent block depends on the number of channels produced by the first convolutional layer.

The hyperparameter optimization process required 100 experiments, namely 100 combinations of hyperparameters were randomly sampled from the search space. To jointly exploit both massive parallelism and aggressive early stopping, we used Asynchronous Successive Halving Algorithm (ASHA)^75^ as a method of parametric optimization. Each trial is trained for a minimum of 2 epochs and a maximum of 50, with a reduction factor of 2. Each random combination of hyperparameters is trained on the training set—after applying random horizontal and vertical flipping—with the goal of minimizing the Cross-Entropy loss function with mini-batch gradient descent using the Adam optimizer. The chosen network is the one that maximizes the F1-Score metric on the validation set.

The best of the randomly sampled configurations was the following: *C*_*Out*_ = 64, *k* = 11, *s* = 2, *p* = 1, *d* = 2, with *lr* = 0.0001 and *B* = 32.

## Supplementary Information

Supplementary Information.

## Data Availability

The INGV seismic data are accessible from the EIDA database (European Integrated Data Archive) at http://eida.rm.ingv.it/. The large DAS dataset is archived at INGV-OE and GFZ and is available upon request to the corresponding author.

## References

[1] Neuberg, J., Luckett, R., Ripepe, M. & Braun, T. Highlights from a seismic broadband array on Stromboli Volcano. *Geophys. Res. Lett.* **21,**10.1029/94GL00377. issn: 0094–8276 (1994).

[2] Chouet, B. A. Seismic Model for the Source of Long-Period Events and Harmonic Tremor. In: Gasparini, P., Scarpa, R., Aki, K. (eds) *Volcanic Seismology*. IAVCEI Proceedings in Volcanology, **vol 3**. Springer, Berlin, Heidelberg. 10.1007/978-3-642-77008-1_11 (1992).

[3] Jousset P, Neuberg J, Jolly A (2004). Modelling low-frequency volcanic earthquakes in a viscoelastic medium with topography. Geophys. J. Int..

[4] Syahbana D (2014). Fluid dynamics beneath a wet volcano inferred from the complex frequencies of long-period (LP) events: An example from Papandayan volcano, West Java, Indonesia during the 2011 seismic unrest. J. Volcanol. Geothermal. Res..

[5] Jousset, P. *et al.* Fibre optic distributed acoustic sensing of volcanic events. *Nat. Commun.* **13,** 1753. 10.1038/s41467-022-29184-w (2022). 10.1038/s41467-022-29184-w PMC8971480 35361757

[6] Harrington, R. M., & Brodsky, E. E. Volcanic hybrid earthquakes that are brittle-failure events. *Geophys. Res. Lett*., **34**, L06308. 10.1029/2006GL028714 (2007)

[7] Bean CJ (2014). Long-Period seismicity in the shallow volcanic edifice formed from slow-rupture earthquakes. Nat. Geosci..

[8] Rowley, P., Benson, P.M. & Bean, C.J. Deformation-controlled long-period seismicity in low-cohesion volcanic sediments. *Nat. Geosci.* **14**, 942–948. 10.1038/s41561-021-00844-8 (2021).

[9] McNutt, S. R., *et al.* The Encyclopedia of Volcanoes, (ed. Sigurdsson, H.), 2nd Edn. (Academic Press (2015).

[10] Giudicepietro, F., *et al.* Geophysical precursors of the July-August 2019 paroxysmal eruptive phase and their implications for Stromboli volcano (Italy) monitoring. *Sci. Rep.* **10**, 10296. 10.1038/s41598-020-67220-1 (2020). 10.1038/s41598-020-67220-1 PMC7314781 32581259

[11] Jousset P (2010). Signs of magma ascent in LP and VLP seismic events and link to degassing: an example from the explosive eruption at Merapi volcano, Indonesia. J. Volcanol. Geotherm. Res..

[12] Park, I., *et al.* Classification of long-term very long period (VLP) volcanic earthquakes at Whakaari/White Island volcano, New Zealand. *Earth Planets Space* **72**, 92. 10.1186/s40623-020-01224-z (2020).

[13] Chouet B, Matoza RS (2013). A multi-decadal view of seismic methods for detecting precursors of magma movement and eruption. J. Volcanol. Geotherm. Res..

[14] Matoza, R.S. & Roman, D.C. One-hundred-year advances in volcano seismology and acoustics. *Bull. Volcanol.* **84**, 1. 10.1007/s00445-022-01586 (2022).

[15] Hansen, S. M. & Schmandt, B. Automated detection and location of microseismicity at Mount St. Helens with a large-N geophone array. *Geophys. Res. Lett.* **42**, 7390–7397 (2015).

[16] Jousset P (2013). Signs of magma ascent in LP and VLP seismic events and link to degassing: An example from the 2010 explosive eruption of Merapi volcano Indonesia. J. Volcanol. Geothermal. Res..

[17] Witze, A. Why the Tongan eruption will go down in the history of volcanology. *Nature* **602**, 376–378. 10.1038/d41586-022-00394-y (2022). 10.1038/d41586-022-00394-y 35140378

[18] Blanck, H., Jousset, P., Hersir, G. P., Ágústsson, K. & Flóvenz, Ó. G. Analysis of 2014–2015 on- and off-shore passive seismic data on the Reykjanes Peninsula, SW Iceland. *J. Volcanol. Geothermal. Res.*, **391**, 106548. 10.1016/j.jvolgeores.2019.02.001 (2020).

[19] Jousset P (2018). Dynamic strain determination using fibre-optic cables allows imaging of seismological and structural features. Nat. Comm..

[20] Currenti G (2021). On the comparison of strain measurements from fibre optics with a dense seismometer array at Etna volcano (Italy). Solid Earth.

[21] Klaasen, S. *et al.* Distributed acoustic sensing in volcano-glacial environments—Mount Meager, British Columbia. *J. Geophys. Res. Solid Earth* **126**, e2021JB022358. 10.1029/2021JB022358 (2021).

[22] Lindsey, N. J., Craig, T. D. & Ajo-Franklin, J. B. Illuminating seafloor faults and ocean dynamics with dark fiber distributed acoustic sensing. *Science* **366**, 1103–1107. 10.1126/science.aay5881 (2019). 10.1126/science.aay5881 31780553

[23] Mousavi, S. M., & Beroza, G. C. Deep-learning seismology. *Science*, **377**(6607). 10.1126/science.abm4470 (2022). 10.1126/science.abm4470 35951699

[24] Baldi, P. Deep learning in science. Cambridge University Press, ISBN: 9781108955652, (2021).

[25] Camps-Valls, G., Tuia, D., Zhu, X. X., & Reichstein, M. (Eds.). Deep learning for the Earth Sciences: A comprehensive approach to remote sensing, climate science and geosciences. John Wiley & Sons. ISBN: 9781119646143 (2021).

[26] Malfante M (2018). Machine learning for volcano-seismic signals: Challenges and perspectives. IEEE Signal Process. Mag..

[27] Titos, M., Bueno, A., García, L., & Benítez, C. A deep neural networks approach to automatic recognition systems for volcano-seismic events. *IEEE J. Select. Top. Appl. Earth Obs. Remote Sens.* **11**(5), 1533–1544. 10.1109/JSTARS.2018.2803198.27 (2018).

[28] Titos M, Bueno A, García L, Benítez MC, Ibañez J (2018). Detection and classification of continuous volcano-seismic signals with recurrent neural networks. IEEE Trans. Geosci. Remote Sens..

[29] Lara F, Lara-Cueva R, Larco JC, Carrera EV, León R (2021). A deep learning approach for automatic recognition of seismo-volcanic events at the Cotopaxi volcano. J. Volcanol. Geothermal Res..

[30] Almendros J, Chouet B (2003). Performance of the radial semblance method for the location of very long period volcanic signals. Bull. Seismol. Soc. Am..

[31] Inguaggiato, S. *et al.* The extensive parameters as a tool to monitoring the volcanic activity: The case study of Vulcano Island (Italy) *Remote Sens*. **14**(5), 1283. 10.3390/rs14051283 (2022).

[32] INGV Report (2022). Bollettino settimanale sul monitoraggio vulcanico, geochimico e sismico del vulcano Vulcano. Available at. https://www.ct.ingv.it/index.php/monitoraggio-e-sorveglianza/prodotti-del-monitoraggio/bollettini-settimanali-multidisciplinari/.

[33] Federico, C., *et al*. Inferences on the 2021 ongoing volcanic unrest at Vulcano Island (Italy) through a comprehensive multidisciplinary surveillance network accepted in Remote Sens. (2023).

[34] Ajo-Franklin JB (2019). Distributed acoustic sensing using dark fiber for near-surface characterization and broadband seismic event detection. Sci. Rep..

[35] Martuganova E (2022). 3D deep geothermal reservoir imaging with wireline distributed acoustic sensing in two boreholes. Solid Earth.

[36] Sladen A (2019). Distributed sensing of earthquakes and ocean-solid Earth interactions on seafloor telecom cables. Nat. Commun..

[37] Williams EF (2019). Distributed sensing of microseisms and teleseisms with submarine dark fibers. Nat. Commun..

[38] Matsumoto H (2021). Detection of hydroacoustic signals on a fiber-optic submarine cable. Sci. Rep..

[39] Ugalde A (2021). Noise levels and signals observed on submarine fibers in the Canary Islands using DAS. Seismol. Res. Lett..

[40] Keller J (1980). The Island of Vulcano. Rendiconti della Società Italiana di Mineralogia e Petrologia.

[41] De Astis, G., *et al.* Geology, volcanic history and petrology of Vulcano (central Aeolian archipelago). *Geol. Soc. Lond. *(**1**), 281–349. Memoirs 37 (2013).

[42] Selva J, *et al.* Multiple hazards and paths to eruptions: A review of the volcanic system of Vulcano (Aeolian Islands, Italy). *Earth Sci. Rev.* **207**, 103186. 10.1016/j.earscirev.2020.103186 (2020).

[43] Bean C, Lokmer I, Obrien G (2008). Influence of near-surface volcanic structure on long-period seismic signals and on moment tensor inversions: Simulated examples from Mount Etna. J. Geophys. Res.: Solid Earth.

[44] Cesca, S., *et al.* Effects of topography and crustal heterogeneities on the source estimation of LP event at Kilauea volcano. *Geophys. J. Int.* **172**, 1219–1236. 10.1111/j.1365246X.2007.03695.x (2008).

[45] O’Brien GS, Bean CJ (2009). Volcano topography, structure and intrinsic attenuation: Their relative influences on a simulated 3D visco-elastic wavefield. J. Volcanol. Geotherm. Res..

[46] Ripperger J, Igel H, Wasserman J (2003). Seismic wave simulation in the presence of real volcano topography. J. Volcanol. Geotherm. Res..

[47] Clarke, J., Adam, L. & vanWijk, K. LP or VT signals? How intrinsic attenuation influences volcano seismic signatures constrained by Whakaari volcano parameters. *J. Volcanol. Geotherm. Res*. 10.1016/j.jvolgeores.2021.107337 (2021).

[48] Martin, E. Passive imaging and characterization of the subsurface with distributed acoustic sensing. Ph.D. Thesis, Stanford Univ., Stanford, CA (2018).

[49] Legrand D, Kaneshima S, Kawakatsua H (2000). Moment tensor analysis of near-field broadband waveforms observed at Aso Volcano, Japan. J. Volcanol. Geotherm. Res..

[50] Kawakatsu H (2000). Aso94: Aso seismic observation with broadband instruments. J. Volcanol. Geotherm. Res..

[51] Cannata, A., *et al.* Monitoring seismo-volcanic and infrasonic signals at volcanoes: Mt. Etna Case Study. *Pure Appl. Geophys*. **170**, 1751–1771. 10.1007/s00024-012-0634-x (2013).

[52] Kohavi, R. & Provost, F. Glossary of terms: Machine learning—special issue on applications of machine learning and the knowledge discovery process. *Mach. Learn.* **30**, 271–274. 10.1023/A:1017181826899 (1998)

[53] Hossin M, Sulaiman MN (2015). A review on evaluation metrics for data classification evaluations. Int. J. Data Min. Knowl. Manag. Process.

[54] Li ZR (2021). ridgecrest earthquake with distributed acoustic sensing. AGU Adv..

[55] Tejedor J (2017). Machine learning methods for pipeline surveillance systems based on distributed acoustic sensing: A review. Appl. Sci..

[56] Shiloh L, Eyal A, Giryes R (2019). Efficient processing of distributed acoustic sensing data using a deep learning approach. J. Lightwave Technol..

[57] Bublin M (2021). Event detection for distributed acoustic sensing: combining knowledge-based, classical machine learning, and deep learning approaches. Sensors.

[58] Wu H (2021). Pattern recognition in distributed fiber-optic acoustic sensor using an intensity and phase stacked convolutional neural network with data augmentation. Opt. Express.

[59] Jayawickrema U (2022). Fibre-optic sensor and deep learning-based structural health monitoring systems for civil structures: A review. Measurement.

[60] Huot F (2022). Detection and characterization of microseismic events from fiber-optic DAS data using deep learning. Seismol. Res. Lett..

[61] Press W, Flannery B, Teukolsky S, Vetterking W (1986). Numerical recipes.

[62] Niu J, Song T-RA (2021). The response of repetitive very-long-period seismic signals at Aso volcano to periodic loading. Geophys. Res. Lett..

[63] Jolly AD, Neuberg J, Jousset P, Sherburn S (2012). A new source process for evolving repetitious earthquakes at Ngauruhoe volcano New Zealand. J. Volcanol. Geotherm. Res..

[64] Vargas, C. *et al.* Breathing of the Nevado del Ruiz volcano reservoir, Colombia, inferred from repeated seismic tomography. *Sci. Rep.* **7**, 46094. 10.1038/srep46094 (2017) 10.1038/srep46094 PMC5385870 28393851

[65] Robin C (2018). Breathing and coughing: The extraordinarily high degassing of Popocatépetl volcano investigated with an SO2 camera. Front. Earth Sci..

[66] Szegedy, C. *et al.* Going deeper with convolutions. arXiv. http://arxiv.org/abs/1409.4842 (2014).

[67] Szegedy, C. *et al.* Rethinking the inception architecture for computer vision. arXiv. http://arxiv.org/abs/1512.00567 (2015).

[68] Szegedy, C., Ioffe, S., Vanhoucke, V., & Alemi, A. Inception-v4, Inception-ResNet and the Impact of Residual Connections on Learning. arXiv. https://arxiv.org/abs/1602.07261 (2016).

[69] Russakovsky O (2015). Imagenet large scale visual recognition challenge. Int. J. Comput. Vision.

[70] Paszke, A., *et al.* Pytorch: An imperative style, high-performance deep learning library. *Adv. Neural Inf. Process. Syst.* **32**. (2019).

[71] Murphy, K. P. *Machine learning: A probabilistic perspective* (MIT Press, 2012).

[72] Kingma, D. P., & Ba, J. *Adam: A Method for Stochastic Optimization*. arXiv. https://arxiv.org/abs/1412.6980 (2014).

[73] Goodfellow, I., Bengio, Y., & Courville, A. *Deep learning* (MIT press, 2016).

[74] Bishop, C. M. *Pattern recognition and machine learning* (4, p. 198) (Springer, New York, 2006).

[75] Li Z, Shen A, Zhan Z (2018). Pushing the limit of earthquake detection with distributed acoustic sensing and template matching: A case study at the Brady geothermal field. Geophys. J. Int..

