# Supplementary material for: Distributed dynamic strain sensing of very long period and long period events on telecom fiber-optic cables at Vulcano, Italy
Source: Sci Rep. 2023 Mar 21;13:4641. doi: 10.1038/s41598-023-31779-2 (PMC10030969; doi:10.1038/s41598-023-31779-2)
Supplement: Supplementary file 1 — Supplementary Information. [file 41598_2023_31779_MOESM1_ESM.docx]

**Supplementary Information**

**Distributed Dynamic Strain Sensing of Very Long Period and Long Period Events on Telecom fiber-optic cables at Vulcano, Italy**

*Gilda Currenti^1^, Martina Allegra^1,2^, Flavio Cannavò^1^, Philippe Jousset^3^,*

*Michele Prestifilippo^1^, Rosalba Napoli^1^, Mariangela Sciotto^1^,*

*Giuseppe Di Grazia^1^, Eugenio Privitera^1,^ Simone Palazzo^2^, Charlotte Krawczyk^3^*

*^1^Istituto Nazionale di Geofisica e Vulcanologia-Osservatorio Etneo, Piazza Roma 2, Catania, Italy*

*^2^Department of Electrical, Electronic and Computer Engineering, University of Catania, Viale Andrea Doria, 6, Catania, 95125, Italy*

*^3^GFZ German Research Centre for Geosciences, Einsteinstrasse 42-46, Potsdam 14473, Germany*

Corresponding author: (gilda.currenti@ingv.it)

**Supplementary Text**

Vulcano Island has a long eruptive history characterized by alternation of Strombolian and effusive activities, Vulcanian, phreatomagmatic and explosive sustained eruptions, separated by periods of quiescence^1^. The latest eruption occurred between 1888 and 1890. Since then, the activity consists mainly of hydrothermal manifestations and degassing with variable intensity from solfataric and fumarolic systems located in the area of La Fossa crater and Vulcano Porto ^2^. After a main crisis in 1988-1991, several unrest phases occurred in the last 30 years, e.g. 1996-98, 2004-05, 2009 and other minor episodes since 2016 ^1,3^. They were generally characterized by increases in emission temperature and flux of fumarolic fluids at the crater, accompanied by an expansion of the exhalative areas and an increase of the magmatic species (CO_2_, He, SO_2_) in crater fumaroles. Concurrently to those geochemical anomalies, increased rate of low magnitude seismo-volcanic events (historically called microseismicity) below La Fossa cone at an average depth of about 500-1500 m bsl has been observed ^4,5,6,7^. These seismic events were characterized by no distinct P and S phases. Based upon time-frequency analysis, three main groups of events were recognized^6,8^: (i) the long period (LP) events whose frequency ranges between 0.5 and 5 Hz and which include hybrid (HB) and mixed (MX) events; (ii) the high frequency (HF) events with a frequency range generally from 5 to 25 Hz, and (iii) monochromatic (MC) events characterized by a single frequency peak at 6 or 8 Hz. Moreover, tornillos, with sharp spectral peaks above 5 Hz are recorded at Vulcano^8^. Their source mechanisms have been attributed to fluid-filled crack or conduit resonance phenomena and/or to fracturing processes occurring in hydrothermally altered rocks ^6, 10^.

**Supplementary Figures**

**
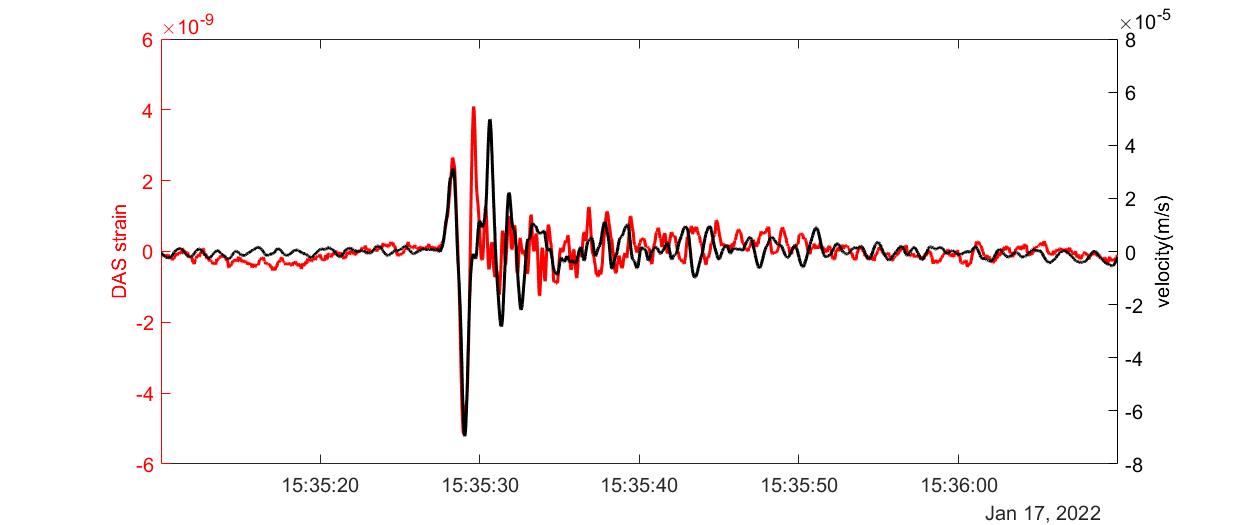
**

**Figure S1** – Common conversion approach^10,11^ to relate strain and velocity of a co-located seismometer by the basic relation ε=vp, where ε is the DAS strain, v the velocity projected along the local fiber direction and p the apparent phase slowness. The comparison is performed between the seismic velocity at OEM5 and the DAS signal at the nearby channel 888, where the highest cross-correlation with seismic signal is achieved. DAS strainrate data is integrated over time to obtain the strain signal. Both seismic velocity and strain signals are then low-pass filtered below 5.5 Hz. The small value of the apparent phase slowness (on the order of 10^-4^ s/m) is indicative of a weak response of the DAS because of coupling conditions and/or cable geometry with respect to the source.


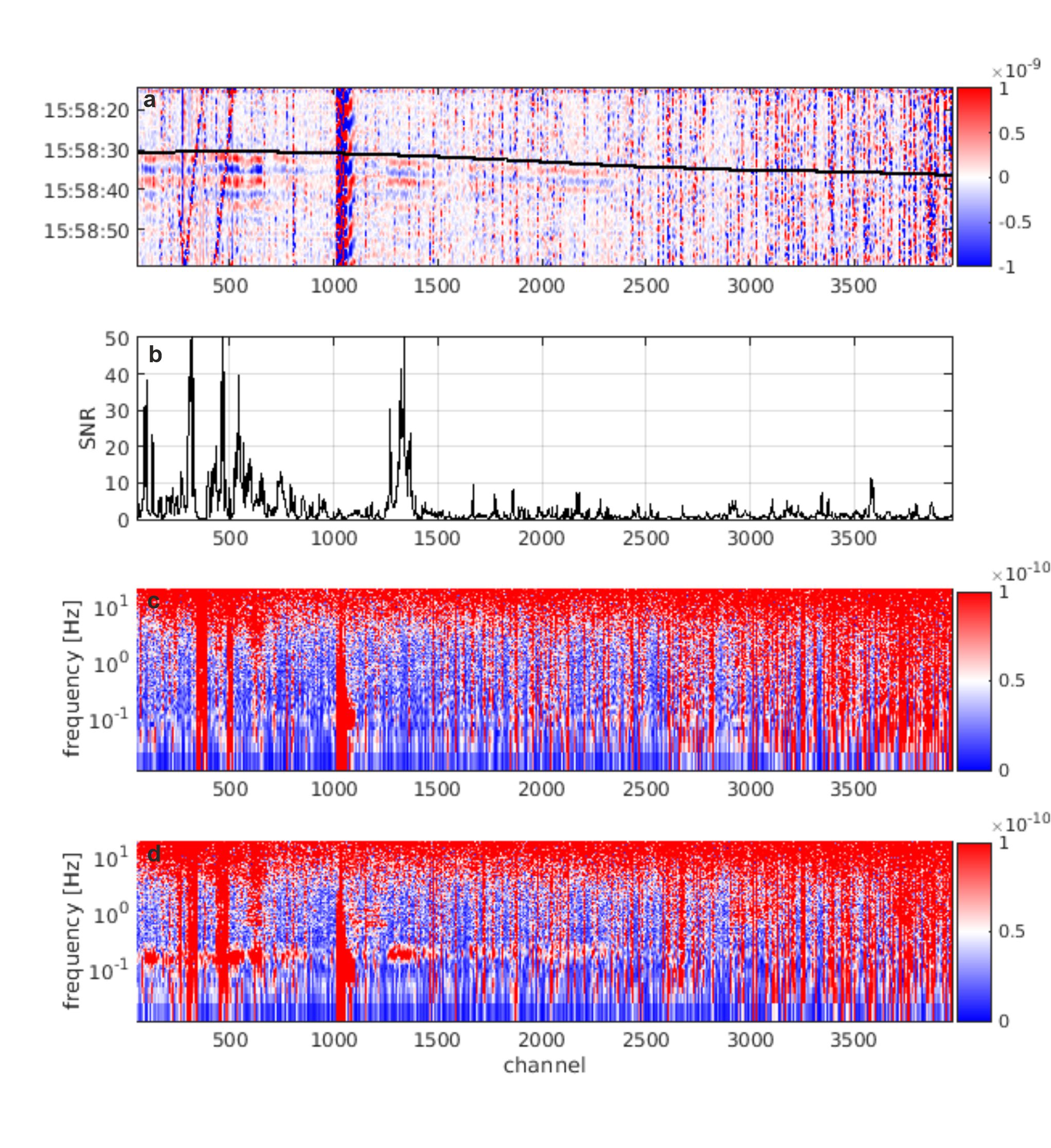


**Figure S2** – SNR calculation. (a) DAS signal filtered below 1 Hz and arrival time at the channels (black line). (b) Time-domain SNR, based on root-mean-square (RMS) values for signal and noise, in 20 s windows selected before and after the arrival time (black line). Spectrum of the noise (c) and of the signal (d) in the time windows before and after the arrival time, respectively.


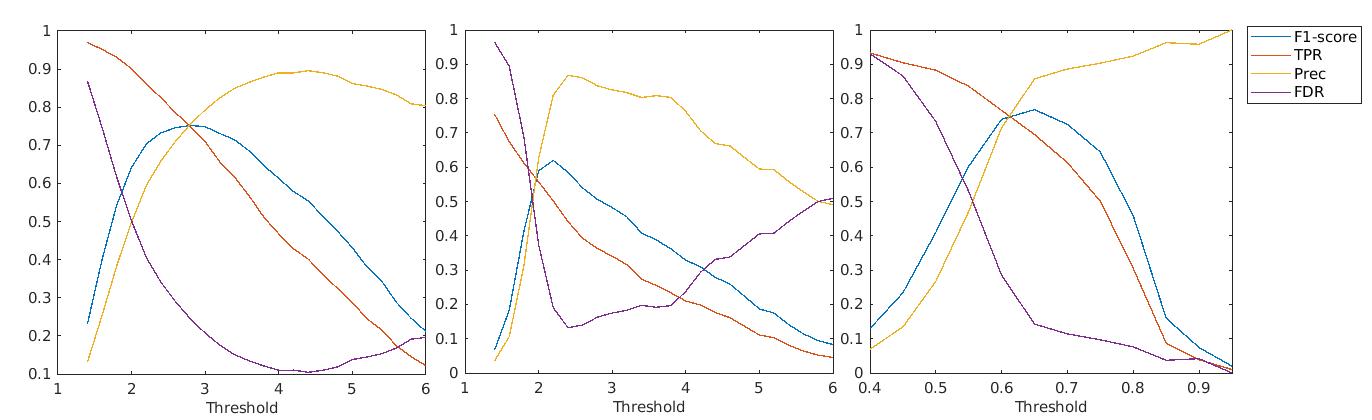


**Figure S3** – Metrics values (see definition in Tab. 1) on the TP, FP, FN events detected using STA-LTA algorithm on the LP (left) and VLP bands (central) for several thresholds. A similar performance analysis is carried out on the 2D template matching detection (right) run on the VLP band.


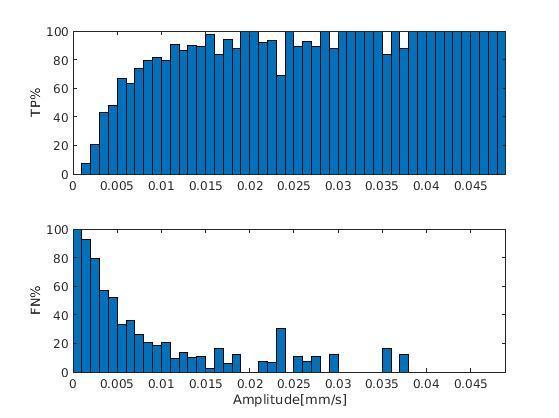


**Figure S4** - Amplitude distribution of the percentage of TP and FN events detected in the DAS records by the STA-LTA algorithm.

**
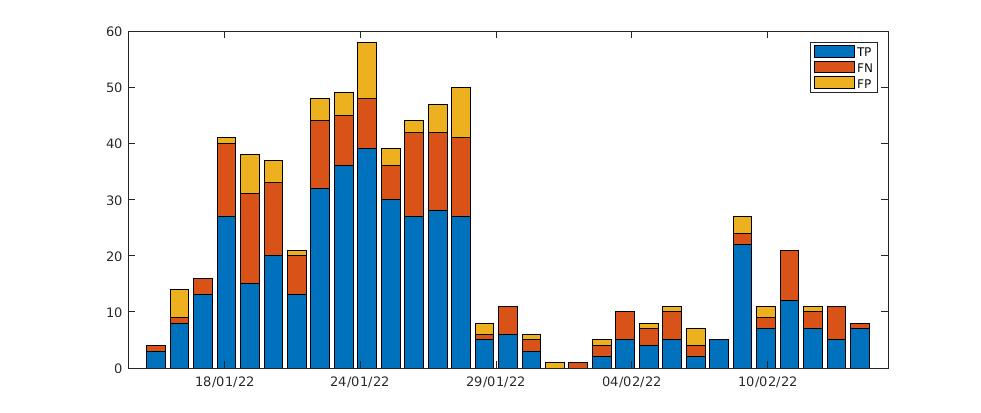
**

**Figure S5** - Distribution of daily VLP events detected in the DAS (TP+FP) data using a 2D template matching detection and compared to the VLP events detected by the INGV seismic network (TP+FN).


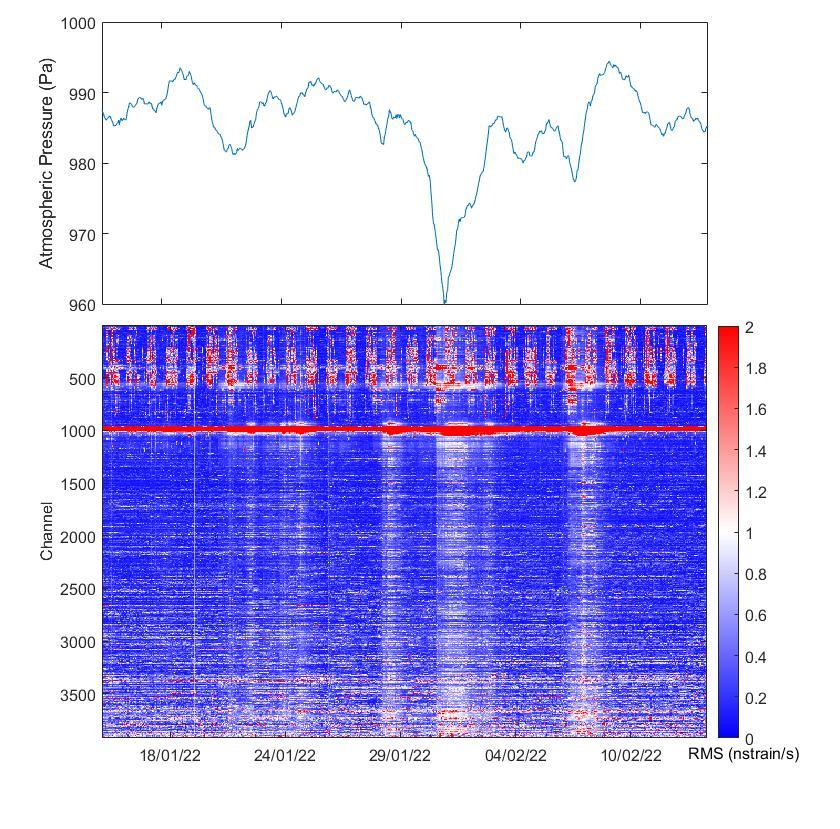


**Figure S6** – (top) Atmospheric pressure recorded at Salina, the eolian station of the SIAS (Servizio Informativo Agrometeorologico Siciliano) network, and (bottom) RMS (Root Mean Square) of the DAS signal in the microseism frequency band (0.05-0.5 Hz). Increase of the microseism RMS is observed during bad weather conditions.

**References**

1. Selva J, et al. A Multiple hazards and paths to eruptions: A review of the volcanic system of Vulcano (Aeolian Islands, Italy). Earth-Science Reviews 207:103186. https://doi.org/10.1016/j.earscirev.2020.103186 (2020)

2. Paonita, A. et al. The Episodic and Abrupt Geochemical Changes at La Fossa Fumaroles (Vulcano Island, Italy) and Related Constraints on the Dynamics, Structure, and Compositions of the Magmatic System. Geochim. Cosmochim. Acta 120, 158–178 (2013).

3. Inguaggiato, S. et al The Extensive Parameters as a Tool to Monitoring the Volcanic Activity: The Case Study of Vulcano Island (Italy) Remote Sens., 14(5), 1283; https://doi.org/10.3390/rs14051283 (2022).

4. Chiodini, G., et al. Geochemical and seismological investigations at Vulcano (Aeolian islands) during 1978–1989. J. Geophys. Res. 445 97:11025–11032 (1992).

5. Aubert, M., Diliberto, S., Finizola, A., Chébli, Y., Double origin of hydrothermal convective flux variations in the Fossa of Vulcano (Italy). Bull. Volcanol. 70:743–751, DOI 419 10.1007/s00445-007-0165-y (2008).

6. Alparone, S. et al. Timespace variation of volcano-seismic events at La Fossa (Vulcano, Aeolian Islands,Italy): new insights into seismic sources in a hydrothermal system. Bull Volcanol 72,803–816 (2010).

7. Alparone S, et al Dynamics of Vulcano Islannad (Tyrrhenian Sea, Italy) investigated by long-term (40 yearras) geophysical data. Earth Sci. Rev. 190, 521-535. https://doi.org/10.1016/j.earscirev.2019.01.002 (2019).

8. Milluzzo V, et al. Tornillos at Vulcano: Clues to the dynamics of the hydrothermal system. J. Volcanol. Geotherm. Res. 198(3-4), 377–393. https://doi.org/10.1016/j.jvolgeores.2010.09.022 (2010).

9. Cannata A, et al. Multiparametric Approach in Investigating Volcano-Hydrothermal Systems: the Case Study of Vulcano (Aeolian Islands, Italy). Pure Appl. Geophys. 169:167–182. https://doi.org/10.1007/s00024-011-0297-z (2012).

10. Currenti, G. et al. On the comparison of strain measurements from fibre optics with a dense seismometer array at Etna volcano (Italy). Solid Earth, 12, 993–1003, https://doi.org/10.5194/se-12-993-2021 (2021).

11. Yu, C., Zhan, Z., Lindsey, N. J., Ajo‐Franklin, J. B., & Robertson, M. The potential of DAS in teleseismic studies: Insights from the Goldstone experiment. Geophysical Research Letters, 46, 1320–1328. https://doi.org/10.1029/2018GL081195 (2019).
